# Special Analytical and Health Commission on Tinned and Preserved Food

**Published:** 1906-09-22

**Authors:** 


### SPECIAL ANALYTICAL AND HEALTH COMMISSION ON TINNED AND PRESERVED FOOD.

### XI. PRESERVED FRUITS.

### Æsthetic Properties of Fruit.

Although the actual nutritive value of fruit is greatly increased when it is made into jam, yet nevertheless fruit in jam never possesses, however carefully the jam may be made, quite the flavour of fresh fruit. Fruit has not only an interest from the point of view of the person who eats it, but also from the point of view of the plant that produces it. Although the attractive qualities, including under this term both appearance and taste, have been enormously increased by artificial culture, yet nevertheless even in its purely natural condition it is absolutely essential to the plant, if it is to succeed in the struggle for existence, to produce fruit which is attractive. The plant's object in producing fruit is in most cases to attract birds and insects, whose function it is to disseminate the seeds of the respective plant. We find that the predominant colours of all fruit are yellow, red, and purple, and these colours are peculiarly associated with sensations of pleasure; the uneducated child will endeavour to grasp and to possess especially red and yellow objects, whereas he will make no effort to obtain blue or grey ones. Hence fruit must be regarded, by virtue of its lusciousness, rather as an agent that increases our appetite, by appealing to our æsthetic sense through its appearance and taste than as an actual nutriment. Its intimate relation, by virtue of the sugar which it contains, to the making of wine gives it, no doubt, additionally a unique place, and in this connection it will probably be remembered that grapes were one of the most cherished trophies brought back by the scouts sent to explore the Promised Land; the luscious fruit stimulated the Israelites to effort, for " to the land of Canaan they set out and to the land of Canaan they came."

*Chemical and Physiological Properties of Fruit.*—Roughly speaking, fruits in their natural state contain about 80 per cent. to 90 per cent. of water. The remainder of their substance is made up chiefly of carbo-hydrates, and of this carbo-hydrate the greater part consists of sugar. The sugar contained in fruit is of a special type, and is termed by chemists lævulose, or fruit-sugar. Some ripe fruit, however, contains also cane-sugar, and some—for instance, grapes—contain dextrose, or grape-sugar. There is evidence to show that lævulose is better assimilated by patients suffering from diabetes or glycosuria than dextrose, and hence in some cases of diabetes one can allow a certain amount of fruit to be taken without increasing the sugar in the urine. An average amount of sugar in fruits is 10 per cent. In addition to these substances fruit contains a certain amount of vegetable gum, and it is this gum which makes fruit set upon prolonged cooking, and is generally known as vegetable jelly. The flavour of fruit is due for the most part to volatile bodies which are termed by chemists compound ethers. Some fruits, however, owe their flavour to the presence of one or more volatile oils. The two remaining important constituents of fruit from the dietetic and medicinal standpoint are acidity, due to vegetable acids and acid salts, and mineral constituents. It is the acidity of fruit that is very apt to disagree with delicate digestions, and unfortunately the taste of sharpness on the tongue when fruit is consumed is not really an index of its acidity. Fruits contain in varying quantities mucilaginous material, and the sharp taste of fruit varies not only directly with the degree of acidity, but also inversely with the amount of the former material present. For instance, nobody would deny that a ripe red-currant tastes tarter and more acid than a ripe raspberry, and yet the acidity of both is the same. Fruits vary considerably in acidity, gooseberries, currants, raspberries, and strawberries being the most acid; ripe pears and ripe peaches the least acid. The mineral constituents of fruit are of considerable importance medicinally. They consist for the most part of potassium salts, of citric, malic, and tartaric acids, and it is to these salts and the sugar they contain that fruits owe their reputation as laxatives. This action is to some extent also increased by the fact that most fruits leave a considerable indigestible residue. This residue, consisting to some extent of the indigestible carbohydrate cellulose, is reduced by cooking; and thus, speaking generally, cooked fruit is more digestible and less laxative than fresh fruit. Certain fruits contain special substances, and amongst these we may mention the pineapple and the cherry. The pineapple contains a digestive ferment, a substance capable of converting proteids into peptones. An enzime, in fact, that acts exactly like pepsine. The cherry contains prussic acid, a substance which has a sedative action upon the stomach.

### Samples Received.

Through the kindness of several manufacturers we have received samples of bottled and preserved fruits. The Kent Jam Company, Limited, of Swanley, Kent, whose jams we noticed in our last article, have sent us a glass bottle of their preserved cherries and one of their preserved gooseberries. These preserved fruits are ready for cooking, and when employed for making tarts and puddings must be sweetened to taste. When treated in this way they possess an excellent appearance and flavour, and, indeed, it would be difficult to tell them from fresh fruit. Messrs. Chivers and Sons, of Histon, Cambridge, have submitted a can of their strawberries and raspberries, each preserved in syrup. These products are exceedingly elegant, and are in the style of the well-known French conserves. The fruit used for them is evidently carefully selected and quite fresh, and this product makes a most delicious and wholesome adjunct to milk puddings, custards, and blanc manges. Messrs. Richard Dickeson and Company have sent us four cans of their preserved fruits as supplied to army canteens and other consumers. The samples sent are pears, apricots, pineapple, and peaches. These fruits as sent out by Messrs. Dickeson are ready for table use. We have examined each of them carefully, and have come to the conclusion that they are in every way to be recommended.

### Fruit Essences.

As we have mentioned above, in the case of certain patients fruit, chiefly on account of the sugar and the acid it contains, can only be eaten sparingly. There is no doubt, for instance, that the alkalinity of the blood is diminished by the consumption of much fruit. In gouty subjects blood alkalinity is important, as any diminution in it is apt to diminish the blood's solvent power for those relatively insoluble salts the deposition of which in the tissues is often the precursor of an attack of gout. For consumers, who want the flavour of fruit without fruit itself, and also for general culinary purposes where it is wished to impart the flavour of fruit to a relatively large otherwise flavourless mass such as jelly, fruit essences are of great value. The preparation of fruit essences, and indeed of vegetable essences, is a most interesting subject, and one into which we should be glad to enter in detail did the space at our command permit us to do so. At present we must rest content with mentioning two samples of fruit essence which have kindly been sent us by Messrs. Sutton, Sons and Company, of King's Cross, London. The preparations we refer to are essence of lemon and essence of vanilla. Both these essences are exceedingly strong and exceedingly pure; a few drops of each will impart to a relatively large mass the flavour of the respective fruit. They are, in our opinion, free from objectionable constituents of all kinds, are quite wholesome, and thoroughly to be recommended.

Our next article will be devoted to preserved vegetables, and in it we shall have the opportunity of considering the chemical and dietetic properties of these important food-stuffs.

